# *Bacillus subtilis* BS-15 Effectively Improves Plantaricin Production and the Regulatory Biosynthesis in *Lactiplantibacillus plantarum* RX-8

**DOI:** 10.3389/fmicb.2021.772546

**Published:** 2022-01-28

**Authors:** Guorong Liu, Rong Nie, Yangshuo Liu, Xue Li, Jiaojiao Duan, Xu Hao, Yumeng Shan, Jingying Zhang

**Affiliations:** Beijing Advance Innovation Center for Food Nutrition and Human Health, Beijing Laboratory of Food Quality and Safety, Beijing Engineering and Technology Research Center of Food Additives, Beijing Technology and Business University, Beijing, China

**Keywords:** bacteriocin, co-culture, quorum sensing, biopreservation, proteome

## Abstract

Plantaricin is a broad-spectrum bacteriocin produced by *Lactiplantibacillus plantarum* with significant food industry application potential. It was found that the plantaricin production of *L. plantarum* RX-8 was enhanced when co-culturing with *Bacillus subtilis* BS-15. This study, therefore, set out to explore how *B. subtilis* BS-15 induces biosynthesis of plantaricin. The effect of co-culturing with *B. subtilis* BS-15 on cell growth, plantaricin production, quorum-sensing (QS) signal molecule PlnA/autoinducer-2 (AI-2) secretion, as well as plantaricin biosynthesis gene cluster and AI-2 synthesis-associated gene expression, was investigated in bacteriocin-producer *L. plantarum* RX-8. When *L. plantarum* RX-8 and *B. subtilis* BS-15 were co-inoculated in Man–Rogosa–Sharp (MRS) for 20 h at an inoculum ratio of 1:1 (10^6^:10^6^ CFU/ml), the greatest plantaricin output (2,048 AU/ml) was obtained, rising by 32-fold compared with the monoculture of *L. plantarum* RX-8. Additionally, co-culture increased PlnA-inducing activity and AI-2 activity by 8- and 1.14-fold, respectively, over monoculture. RT-qPCR findings generated every 4 h (4–32 h) demonstrated that *B. subtilis* BS-15 remarkably improved the transcription of *plnABCD* and *plnEF*, and increased *pfs* and *luxS* transcription, even when using 200 mM D-ribose, a kind of AI-2 inhibitor. Based on the above findings, co-culturing with *B. subtilis* BS-15 as an environmental stimulus could activate the plantaricin induction *via* the PlnA-mediated intraspecies QS system and the AI-2-mediated interspecies QS system. Moreover, the inducing effect of PlnA and AI-2 in co-culture was independent. Differential proteomics analysis of *B. subtilis* BS-15 in co-culture indicated that bacteriocin-inducing regulatory mechanism may be related to flagellar assembly, peptidoglycan biosynthesis, anaerobic respiration, glycine cleavage system, or thiamin pyrophosphate biosynthesis.

## Introduction

*Lactiplantibacillus plantarum* is a common and versatile lactic acid bacteria (LAB) that is found in a wide variety of fermented foods and human gastrointestinal tract (Corsetti and Valmorri, 2001). In the mixed-culture food fermentations, *L. plantarum* not only contribute significantly to food preservation and fortification, as well as flavors and texture, but also secretes a wide range of beneficial compounds, including bacteriocins (da Silva Sabo et al., 2014), short-chain fatty acids (Osei Sekyere et al., 2020), and polysaccharides (Zhang et al., 2020). Plantaricin is the name given to the bacteriocins produced by *L. plantarum*. Their presence in food is generally considered safe for consumers due to their inactivation by pancreatic or gastric enzymes (Simons et al., 2020). Plantaricin has been found to be efficient against pathogenic and spoilage microorganisms when being used as a food biopreservative ingredient (Zhu et al., 2014; Todorov et al., 2018; Le et al., 2019; Lei et al., 2020). However, industrial production and use of plantaricin are challenging to achieve due to the low production and high cost associated with laboratory culture (Gutiérrez-Cortés et al., 2018). Moreover, the capability of *L. plantarum* to biosynthesize plantaricin may be gradually lost in a noncompetitive environment (Vriezen et al., 2009).

Several strategies were used to enhance plantaricin biosynthesis, including the screening for high-yield strains, optimizing their production conditions, physical and chemical mutagenesis, and heterologous expression. Nevertheless, they have some drawbacks, including inadequate specificity, restricted growth potential, genetic instability, and low safety (Man and Xiang, 2021). Recent research indicates that microbial co-culture fermentations are favorable for bacteriocin production and are extensively used among *L. plantarum* strains that produce bacteriocin (Maldonado-Barragán et al., 2013). For example, in a test of screening bacteriocin-inducing strains under co-culture conditions, 10 out of 45 strains were able to cause *L. plantarum* J23 to produce plantaricin (Rojo-Bezares et al., 2007), 41 out of 82 strains were able to stimulate *L. plantarum* NC8 to produce plantaricin (Maldonado et al., 2004), and 4 out of 76 strains could induce *L. plantarum* KLDS1.0391 to produce plantaricin (Man et al., 2012). Co-culturing with certain bacterial strains as an environmental stimulus may be a feasible technique for improving plantaricin production in the food sector due to the possibility of synergistic exploitation of the metabolic pathways of all engaged strains.

Understanding the mechanism of induction regulation behind bacteriocin production *via* co-culture is crucial for ensuring its effective application in the food industry (Chanos and Mygind, 2016). Until present, only the mechanism of plantaricin biosynthesis in monoculture has been thoroughly researched. As previously documented, plantaricin biosynthesis in monoculture is density-dependently regulated *via* an interspecies quorum-sensing (QS) system mediated by autoinducing peptide (AIP) (Sturme et al., 2007; Diep et al., 2009). The *plnABCD* operon encodes the QS system, which consists of an AIP gene *plnA*, a membrane-located histidine protein kinase (HPK) gene *plnB* and two response regulator (RR) genes *plnC* and *plnD* (Sturme et al., 2007). The AIP, also known as PlnA in *L. plantarum*, is responsible for determining the cell density of its producer (Diep et al., 2009). When PlnA reaches a level detectable by the extracellular part of the histidine kinase (PlnB), PlnB will catalyze the autophosphorylation of the cytoplasmic domain (Meng et al., 2021). Following this, the two RRs phosphorylate and attach to *plnEF*, which encodes the bacteriocin structural genes. Subsequently, the bacteriocin is secreted from the cell *via* the ABC-transport system (Zhao et al., 2021).

However, the bacteriocin-inducing regulation mechanism in co-culture with specific bacteria has been challenging, as both the AIP-mediated interspecies QS system and autoinducer-2 (AI-2)-mediated intraspecies QS system exist simultaneously (Sieuwerts et al., 2008). Some research has demonstrated a positive correlation between the AI-2 activity of *L. plantarum* and bacteriocin production in co-culture (Di Cagno et al., 2009; Man et al., 2012, 2014). Moreover, the HPK and RR interspecies QS system continue to play core roles in the induction in co-culture conditions. In one instance, the PLNC8 was used to stimulate the production of bacteriocins by *L. plantarum* NC8 after co-culture with certain Gram-positive bacteria (Maldonado et al., 2004). When the *plNC8-plNC8HK-plnD* and *plnBCD* genes were removed from *L. plantarum* NC8 and WCFS1, respectively, the potential of these strains to generate bacteriocin through the co-culture with inducing bacterial strains was abolished (Maldonado-Barragán et al., 2013). However, the regulation intensity of AIP-mediated interspecies QS system/AI-2-mediated intraspecies QS system on plantaricin induction and their relationship in co-culture remain unknown.

*Bacillus subtilis* BS-15 was extracted from Chinese traditional cereal vinegar brewing. In previous studies, it was discovered that the co-culture with *B. subtilis* BS-15 had enhanced the plantaricin production of *L. plantarum* RX-8. This breakthrough in plantaricin production makes industrial production and food preservation applications possible (Gutiérrez-Cortés et al., 2018). This study, therefore, set out to explore how *B. subtilis* BS-15 induces biosynthesis of plantaricin. To this end, this paper analyzed the influence of co-culturing with *B. subtilis* BS-15 on the growth of bacteriocin-producer *L. plantarum* RX-8, plantaricin production, QS signal molecule PlnA/AI-2 secretion, as well as plantaricin biosynthesis gene cluster and AI-2 synthesis-related gene expression. Furthermore, the addition of the QS inhibition D-ribose helped assess the effect of AI-2-mediated interspecies QS system on plantaricin production of *L. plantarum* RX-8 in co-culture with *B. subtilis* BS-15. Finally, differential proteomics was used to investigate the changes in the protein level of bacteriocin-inducer *B. subtilis* BS-15 in co-culture.

## Materials and Methods

### Bacterial Strains, Culture Media, and Growth Conditions

The *L. plantarum* RX-8 strain was isolated from the traditional Chinese Sichuan Pao Cai and has been preserved in the China General Microbiological Culture Collection Center (CGMCC 20852). *Listeria monocytogenes* 35152 (ATCC 35152) and *Vibrio harveyi* BB170 (ATCC BAA-1117) were obtained from the American Type Culture Collection (ATCC). The stable bacteriocin-producer *L. plantarum* RX-8 was grown in de Man–Rogosa–Sharp (MRS) broth at 37°C. *B. subtilis* BS-15, isolated from Chinese traditional cereal vinegar brewing, is grown in tryptic soy broth (TSB) at 37°C and preserved in CGMCC (CGMCC 20851). *L. monocytogenes* 35152, grown in TSB at 37°C, was used as the indicator strain to detect the inhibitory and induction activities in bacteriocin production. *V. harveyi* BB170 was a reporter strain to detect the AI-2 activity of co-culture supernatants and grown in marine broth (MB) media at 30°C, 180 rpm. All strains were stored at −80°C in 40% (v/v) glycerol and resuscitated twice in their corresponding broth medium before being used.

### Experiment With Co-culture

A two-compartment separated co-culture system in Transwell plates was used to evaluate the effect of the secretion of *B. subtilis* BS-15 on the plantaricin production of *L. plantarum* RX-8. To be specific, *L. plantarum* RX-8 cells were suspended in co-culture medium and placed in the bottom wells of Transwell plates (Corning, United States), while *B. subtilis* BS-15 cells were placed in the upper wells, separated from the bottom wells by a membrane with a 0.4-μm pore size. To establish the optimal inducible co-culture, two strains were co-inoculated in MRS at different inoculum ratios (Table 1). D-ribose is a kind of competitive inhibitor, which could inhibit AI-2-induced intracellular communication. In order to study the role of AI-2-induced QS regulation system in co-cultures, D-ribose was added, and then the viable cell count, plantaricin production, QS signal molecule activity, as well as bacteriocin biosynthesis gene cluster and AI-2 synthesis-related gene expression of harvested co-culture at pre-fixed incubation times were compared. The monoculture of *L. plantarum* RX-8 was used as control.

**TABLE 1:** Scheme of the inoculation of *L. plantarum* RX-8 and *B. subtilis* BS-15 in co-culture systems with different inoculum ratios.

Treatment	*L. plantarum* RX-8	*B. subtilis* BS-15
1 (control 1)	10^7^	–
2 (A)	10^7^	10^6^
3 (B)	10^7^	10^5^
4 (C)	10^7^	10^4^
5 (control 2)	10^6^	–
6 (D)	10^6^	10^6^
7 (E)	10^6^	10^5^
8 (F)	10^6^	10^4^
9 (control 3)	10^5^	–
10 (G)	10^5^	10^6^
11 (H)	10^5^	10^5^
12 (I)	10^5^	10^4^

### Plantaricin Production Assay

The twofold serial dilution assayed the production level of plantaricin in co-culture/monoculture through agar well diffusion (Zhu et al., 2014). *L. monocytogenes* 35152 (10^7^ CFU/ml) was used as the indicator strain. After a 24-h stationary incubation at 37°C, the cells were removed by centrifugation at 10,000 rpm for 10 min at 4°C to obtain cell-free supernatants (CFSs). Then the 51.6-g ammonium sulfate was added to 100 ml of CFS with continuous stirring by a magnetic stirrer for 1 h. After storing at 4°C overnight, the protein precipitate was pelleted by centrifugation at 12,000 rpm for 10 min at 4°C and solubilized in 20 mM sodium phosphate buffer pH 6.0 (10 ml per 100-ml culture). Then 100 μl of the 10-fold concentrated plantaricin was placed into each well. The plates were held at 4°C for 8 h to allow the bacteriocin to completely diffuse, and these plates were incubated overnight at 37°C. The bacteriocin activity was expressed in arbitrary units (AU/ml), which is represented as reciprocal of the highest dilution showing a distinct zone of inhibition and calculated according to the equation:

$$\text{B}\mathrm{acteriocin}\mathrm{activity}\left(\text{AU}/\text{ml}\right)=\frac{a^{n}}{b\times c}$$

where a is 2 (dilution factor), n is the reciprocal of the highest dilution that resulted in inhibition of the indicator strain, b is 100 μl (sample volume in each well), and c is 10 (sample concentration fold). Furthermore, the relative bacteriocin activity was defined as the ratio of bacteriocin activity in co-culture to that in monoculture. It was used to evaluate the influence of *B. subtilis* BS-15 on plantaricin production in co-culture.

### Analysis of Pln A Activity

#### Culture Preparation for Nonbacteriocin Producing

The nonbacteriocin-producing (Bac^–^) culture is used to analyze the inducing activity of PlnA. Due to the effect of intraspecies QS on bacteriocin production in monoculture, *L. plantarum* loses the ability to produce bacteriocins when inoculated on liquid media with an inoculum size less than the specified threshold (Maldonado-Barragán et al., 2009). To prepare the nonbacteriocin-producing (Bac^–^) culture, the inoculum size of *L. plantarum* RX-8 in monoculture was diluted. Afterward, the 100-ml 24-h-old culture was centrifuged for 2 min at 8,000 rpm and resuspended it in 1 ml of fresh MRS medium after removing the supernatants to get the initial concentration of 1.4 × 10^11^ CFU/ml of *L. plantarum* RX-8 cell suspension. The cell suspension was then serially diluted 10-fold for six times, and 1% (v/v) of each dilution was inoculated in sterile MRS broth to generate the final inoculum size of approximately 10^9^, 10^8^, 10^7^, 10^6^, 10^5^, and 10^4^ CFU/ml, respectively. After incubation at 37°C for 24 h, the samples were centrifuged for 10 min at 10,000 rpm to remove the cells, and the CFS was subsequently measured for plantaricin production using the agar well diffusion method as described above.

#### Semipurified Pln A Activity Assay

PlnA was semipurified according to the method of Maldonado-Barragán et al. (2009) with some modifications. As described above, after a 24-h-old co-culture/monoculture of *L. plantarum* RX-8, cells were removed by centrifugation (10,000 rpm, 10 min), and the pH was adjusted to 7.0. The PlnA present in the supernatant fraction was concentrated by ammonium sulfate precipitation (80% saturation). After the mixture had been stirred overnight at 4°C, the precipitate was pelleted by centrifugation (12,000 rpm, 10 min). The collected precipitate was then dissolved in 20 mM sodium phosphate buffer pH 7.0 and desalted using PD10 gel filtration column (GE Healthcare, WI, United States) equilibrated with the same buffer. The mixture was then applied into a SP-Sepharose Fast Flow cation exchange column (GE Healthcare, WI, United States) equilibrated with 20 mM sodium phosphate buffer (pH 7.0). The fraction with charged cationic property was eluted with a linear salt gradient (0 to 1 M of NaCl) at a flow rate of 1 ml/min and then desalted by a Sephadex G15 column (Sigma-Aldrich, Shanghai, China) preequilibrated with 50 mM sodium acetate buffer pH 5.2. The mixture was further purified by Sephadex G25 column (Sigma-Aldrich, Shanghai, China), and the active fraction was eluted employing the same acetate buffer at a flow rate of 0.5 ml/min. Different fractions were collected and concentrated in a Vacuum Rotary Evaporation Concentrator (Jiaimu, Beijing, China).

The inducing activity tests selected the active fraction containing PlnA. Briefly, each 50-μl concentrated fraction was added to Bac^–^ culture and cultured for 24 h at 37°C. The recovered bacteriocin-producing activity was defined as the PlnA-inducing activity. The relative PlnA-inducing activity was calculated by the ratio of semipurified PlnA-inducing activity in co-culture CFS to that in monoculture CFS.

### Analysis of Autoinducer-2 Activity

The cells in the co-culture/monoculture sample were removed by centrifuging at 10,000 rpm, 4°C, for 10 min. The CFS was filtrated with a 0.22-μm sterile filter and adjusted to pH 7.0. Then the neutralizing CFS was concentrated 10-fold by a Vacuum Rotary Evaporation Concentrator (Jiaimu, China). The reporter strain *V. harveyi* BB170 was diluted 1:5,000 with fresh Autoinducer Bioassay (AB) medium, and the 10-fold concentrated CFS sample was added to the diluted BB170 culture at 1:10 (v/v). The mixture was incubated at 30°C for 4 h with agitation (180 rpm), and 200-μl aliquots were added to white 96-well plates (Thermo, United States) to detect AI-2 activity. The suspension of strain BB170 in AB medium (1:5,000) was used as a blank control. Relative luminescence units (RLU) were measured using a Multi-Detection Plate Reader (SpectraMax i3, Molecular Devices, United States) in luminescence mode. The relative AI-2 activity was defined as the ratio of RLU (the value of CFS)/(the value of blank control), and it was used to evaluate the effect on AI-2 activity of strain RX-8 under different culture conditions.

### Determination of the Concentration of D-Ribose to Be Added

D-ribose inhibition of co-culture AI-2 activity was also determined by using the BB170 reporter strain. D-ribose (0, 10, 50, 100, and 200 mM) was added to the co-culture system and incubated at 37°C for 24 h to find out the appropriate concentration of D-ribose with optimal inhibitory effect. Then as described above, the AI-2 activity of the co-culture sample with D-ribose was detected according to RLU. The co-culture supernatant without D-ribose was used as a control.

### Polymerase Chain Reaction and Quantitative Real-Time Reverse Transcription Polymerase Chain Reaction Analysis of Plantaricin Biosynthesis Gene Clusters and Autoinducer-2 Synthesis-Related Gene Expression

The PCR has tested and determined the existences of *plnA* gene encoding AIP, *plnB* gene encoding HPK, *plnC* and *plnD* gene encoding RRs, AI-2 synthase genes (*pfs* and *luxS*), and bacteriocin structural genes (*plnEF*) in *L. plantarum* RX-8. This study designed the primers based on known sequences of corresponding genes in *L. plantarum* from GenBank (Supplementary Table 1). The following conditions were applied for PCRs: denaturation at 95°C for 5 min, followed by 30 cycles of 94°C for 30 s, annealing at 53°C for 30 s, polymerization at 72°C for 10 s, and final elongation at 72°C for 10 min. The study identified the amplicons obtained by agarose gel electrophoresis.

Quantitative real-time reverse transcription polymerase chain reaction (RT-qPCR) determined the expression level of plantaricin biosynthesis gene clusters (*plnA*, *plnB*, *plnC*, *plnD*, *plnE*, and *plnF*) and AI-2 synthesis-related genes (*pfs* and *luxS*). First, the cells were harvested from monoculture/co-culture and stored in liquid nitrogen for RNA extraction. According to the recommendations of the manufacturer, the total RNA of *L. plantarum* RX-8 were extracted with the RNAprep Pure Cell/Bacteria Kit (Tiangen Biotech, China). The FastQuant RT Kit (with gDNase; Tiangen Biotech, China) synthesized the first-strand cDNA with the FQ-RT Primer Mix. The RT-qPCR monitoring of gene expression was realized by SYBR Green PCR master mix (Tiangen Biotech, China) with the software of CFX96 Real-Time PCR Detection System 3.0 version (Bio-Rad, Hercules, CA, United States). Primer Premier V6.1 (Supplementary Table 2) designed primers for *plnA*, *plnB*, *plnC*, *plnD*, *plnE*, *plnF*, *luxS*, *pfs*, and 16S rRNA genes (NCBI accession No. AL935263). The transcription of total RNA was reversed into cDNA, which was used for qPCR with unigene-specific primers. The amplification program was performed by the SuperReal PreMix Plus (SYBR Green) for the fluorophore SYBR green with fluorescein in a process including 95°C for 5 min, followed by 30 cycles of 94°C for 30 s, 53°C for 30 s, and 72°C for 10 s. The comparative threshold cycle (CT) method calculated the relative abundance of transcripts. The 16S rRNA genes were used as the housekeeping reference gene. RT-qPCR was triplicated for each sample.

### Proteomics Analysis

Both the positive and negative bacteriocin-inducing culture were fermented at 37°C for 20 h, and the fermentation broth was centrifuged at 10,000 rpm for 10 min at 4°C Then the cells of *B. subtilis* BS-15 were sent to Beijing YM Biological Institute (YMBio, Beijing, China) for proteomics analysis, which applied *t*-test and set the *p*-value at 0.05 as the threshold to determine significance in the two groups of regulated proteins. The GengGO/MetaCore software^1^ and KEGG website^2^ analyzed the pathway enrichment with the false discovery rate (FDR) cutoff at 0.001. The Benjamini–Hochberg method adjusted the *p*-values for multiple test corrections. In the GO category and KEGG pathway analysis, the cut-off is less than 0.01.

### Statistical Analysis

All experiments were performed in triplicates. Representative data and images are presented in this paper. The data analysis involved the one-way ANOVA engaged in the Duncan test that used SPSS Statistical package Version 16.0 (SPSS Inc., Chicago, IL, United States). Statistical significance was considered at *p* < 0.05, and the results were expressed as mean ± SD.

## Results

### Construction of Non-bacteriocin-Producing Culture in Monoculture

**FIGURE 1:**
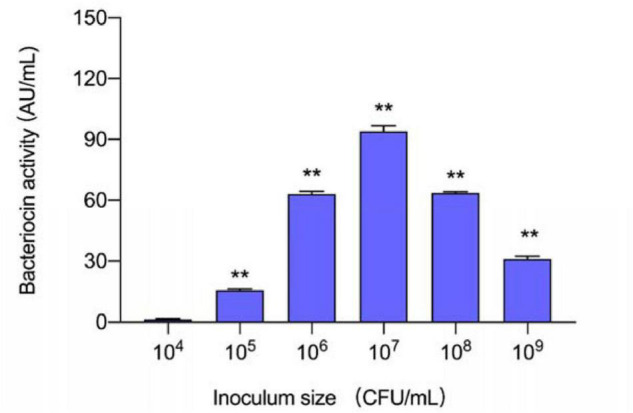
Effect of different inoculum sizes of *L. plantarum* RX-8 on the production of plantaricin. **p < 0.01.

Six inoculum volumes have different effects on the production of plantaricin. As shown in Figure 1, 10^5^–10^9^ CFU/ml had positive influences on plantaricin biosynthesis, while when the volume is 10^4^ CFU/ml, the influences on plantaricin biosynthesis turned to be negative. In addition, *L. plantarum* RX-8 could not produce bacteriocins when being inoculated in liquid media with less than 10^5^ CFU/ml of inoculum, but the production ability would be restored when being added with CFS from a previous bacteriocin-producing (Bac^+^) culture (data not shown). The results in this section showed that the threshold inoculum size was 10^5^ CFU/ml, and the inoculum size of 10^4^ CFU/ml could be used for preparing non-bacteriocin-producing (Bac^–^) culture.

### Effect of Inoculum Ratio on the Production of Plantaricin

**FIGURE 2:**
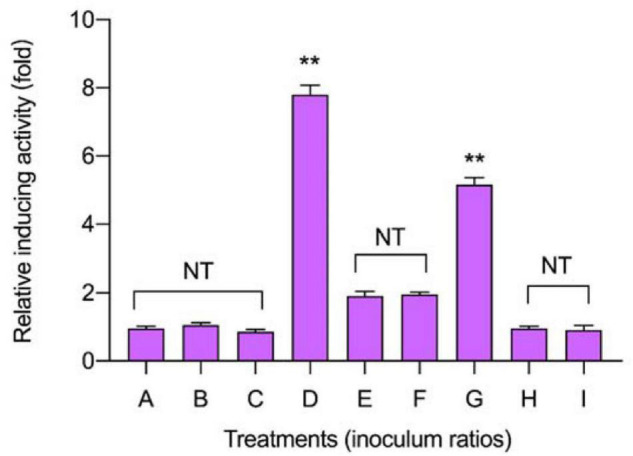
Effects of different inoculum ratios on the production of plantaricin in co-culture. The relative bacteriocin activity was defined as the ratio of bacteriocin activity in co-culture to that in monoculture. The inoculum ratios of A–I are shown in Table 1. NT indicates that there are no significant differences in the results in the control; *p < 0.05 and the **p < 0.01.

To discover the appropriate inducible co-culture, several treatments (A–I) with varying inoculum ratios of *L. plantarum* RX-8 and *B. subtilis* BS-15 were designed to evaluate bacteriocin-inducing activity (Figure 2). The maximum plantaricin output of 1,024 AU/ml was obtained in the co-culture system, which increased by eightfold compared with monoculture (Control 2), due to the 1:1 ratio (10^6^:10^6^ CFU/ml) of *L. plantarum* RX-8 to *B. subtilis* BS-15. For the same inoculum size (10^6^ CFU/ml) of *L. plantarum* RX-8, incubating 10^6^ CFU/ml of *B. subtilis* BS-15 (treatment D) could exert the most positive bacteriocin-inducing activity, which was negative for 10^4^ CFU/ml of *B. subtilis* BS-15 (treatment F). These findings indicate that the appropriate inoculation of *B. subtilis* BS-15 is critical for a robust inducing effect in the co-culture. Due to the apparent disparities between treatments D and F, two treatments were selected for subsequent experiments to assay the relationship between PlnA/AI-2 secretion and plantaricin production. Treatment D, a 1:1 inoculum ratio (10^6^:10^6^ CFU/ml) of *L. plantarum* RX-8 and *B. subtilis* BS-15 in fresh MRS broth at 37°C, was selected as a positive bacteriocin-inducing co-culture (PC). As for treatment F, the co-culture of *L. plantarum* RX-8 and *B. subtilis* BS-15 at a 100:1 inoculum ratio (10^6^:10^4^ CFU/ml) in fresh MRS broth at 37°C was designated as a negative bacteriocin-inducing co-culture (NC).

### Quorum-Sensing Signal Molecule Activity and Plantaricin Production in Co-culture

**FIGURE 3:**
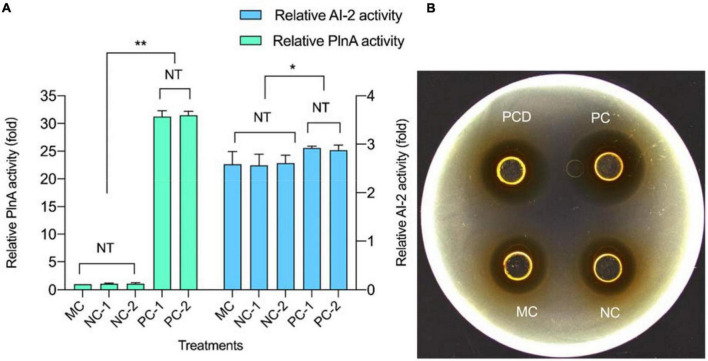
Quorum-sensing (QS) signal molecule secretion and plantaricin production at 24 h in co-culture. **(A)** QS signal molecules. **(B)** Plantaricin production. PC means positive bacteriocin-inducing co-culture of *L. plantarum* RX-8 and *B. subtilis* BS-15 (10^6^:10^6^ CFU/ml) at 37°C for 24 h, PCD means PC with 200 mM D-ribose, and NC means negative bacteriocin-inducing co-culture of *L. plantarum* RX-8 and *B. subtilis* BS-15 (10^6^:10^4^ CFU/ml) at 37°C for 24 h. MC means monoculture of *L. plantarum* RX-8 (10^6^ CFU/ml) at 37°C for 24 h. “1” represents CFS from the upper well, and “2” represents CFS from the bottom well. NT indicates that there were no significant differences in the results between the control; *p < 0.05 and **p < 0.01. The relative AI-2 activity (fold) is defined as (the RLU of CFS)/(the RLU of blank control). The relative PlnA activity (fold) is defined as (the bacteriocin activity of co-culture)/(the bacteriocin activity of monoculture).

The secretion of QS signal molecules PlnA and AI-2 in mono-culture and co-culture (PC and NC) are shown in Figure 3. In co-culture CFS, the activity of the two QS signal molecules composed of one part of *L. plantarum* RX-8 culture CFS (PC-1 and NC-1) and one part of *B. subtilis* BS-15 culture CFS (PC-2 and NC-2) were not significantly different. It was found that both QS signal molecules increased in PC over 24 h, but the secretion remained unchanged in NC. Meanwhile, the production of plantaricin showed a similar rule in PC, NC, and control. According to Figure 3, the activity of QS signal molecules and plantaricin production in PC were higher than those in NC. Besides, there were no significant difference in the activity of two QS signal molecules and plantaricin production. It can be deduced that the QS system engaged in the induction of the production of plantaricin.

### The Determination of D-Ribose Concentrations

**FIGURE 4:**
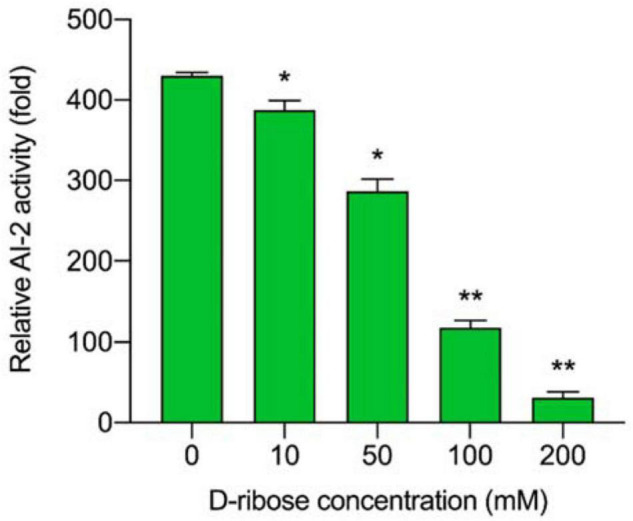
The inhibitory effect of different D-ribose concentrations on AI-2 activity. The relative AI-2 activity is defined as (the RLU of CFS)/(the RLU of blank control). *p < 0.05 and **p < 0.01.

D-ribose bound competitively to the AI-2 transporter (LuxP) or AI-2 receptors (Ryu et al., 2016) of *L. plantarum* RX-8, thus, inhibiting AI-2-induced intracellular communication. As shown in Figure 4, all D-ribose inhibited AI-2 activity regardless of the concentration, whereas 200 mM D-ribose reached more than 90% inhibition (92.76%). Therefore, 200 mM D-ribose was selected for further analysis, and PC with 200 mM D-ribose was defined as PCD.

### Effect of Different Culture Systems on Growth and Bacteriocin Production

**FIGURE 5:**
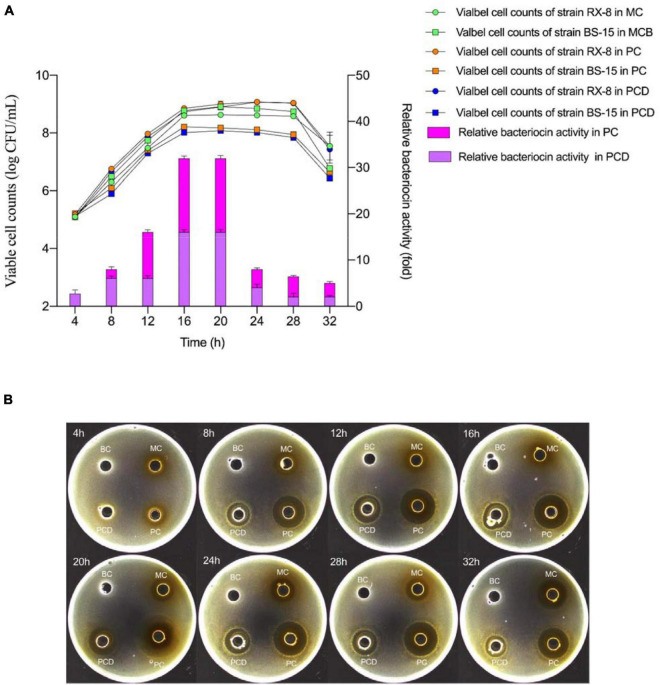
Viable cell amount and plantaricin production in co-culture. **(A)** Viable counts (left) and relative bacteriocin activity (right). **(B)** The plantaricin production at every 4 h from 4 to 32 h. PC means positively bacteriocin-inducing co-culture of *L. plantarum* RX-8 and *B. subtilis* BS-15 (10^6^:10^6^ CFU/ml) at 37°C in 4–32 h. PCD means PC with 200 mM D-ribose. MC means monoculture of *L. plantarum* RX-8 (10^6^ CFU/ml) at 37°C in 4–32 h, MCB means monoculture of *B. subtilis* BS-15 (10^6^ CFU/ml) at 37°C in 4–32 h. BC means blank control (20 mM sodium phosphate buffer, pH 6). The relative bacteriocin activity was defined as the ratio of bacteriocin activity in co-culture to that in monoculture.

The viable cell amount of *L. plantarum* RX-8/*B. subtilis* BS-15 and the relative bacteriocin production in monoculture (MC) and co-culture (PC and PCD) from 4 to 32 h are shown in Figure 5. As shown in Figure 5A, the viable cell counts of *L. plantarum* RX-8 in PC and PCD were increased slightly than in MC, while the viable cell counts of the *B. subtilis* BS-15 in PC and PCD were significantly lower than in MC (*p* < 0.05), which is easy to understand as the plantaricin and other antibacterial substances (e.g., lactic acid) in medium could depress the survival of *B. subtilis* BS-15. The results in Figure 5B illustrated that *L. plantarum* RX-8 began to biosynthesize plantaricin at 8 h in PC and PCD, while the initial production time of plantaricin was 12 h in MC. At the 20th hour, the plantaricin production in all treatments reached the highest point and stabilized in the 20- to 28-h period before decreasing at the 32th hour. Compared with the slight increase in viable cell counts of *L. plantarum* RX-8, the bacteriocin activity of *L. plantarum* RX-8 in PC and PCD was promoted effectively. Specifically, plantaricin production in PC finally reached 2,048 AU/ml at 20 h, 32 times greater than that in MC, and for PCD, the statistics were 1,024 AU/ml and 16 times greater. A certain competitive relationship existed between *L. plantarum* RX-8 and *B. subtilis* BS-15, which may induce the plantaricin production as an environmental stimulus. Meanwhile, after adding AI-2 inhibitor, the reduction of bacteriocin-inducing activity is 25% (8 h), 62.5% (12 h), 50% (16–24 h), 68.75% (28 h), and 60% (32 h), respectively. These results showed that the proportion of regulation intensity of AI-2 on the bacteriocin-inducing activity in the 32-h culture period was variable. It was presumed that the AI-2-mediated intraspecies interspecies QS system played a more important role in late-exponential and stationary phases than that in early-exponential phase.

### Quorum-Sensing Signal Molecule Activity in Positive Bacteriocin-Inducing Co-culture With/Without D-Ribose

**FIGURE 6:**
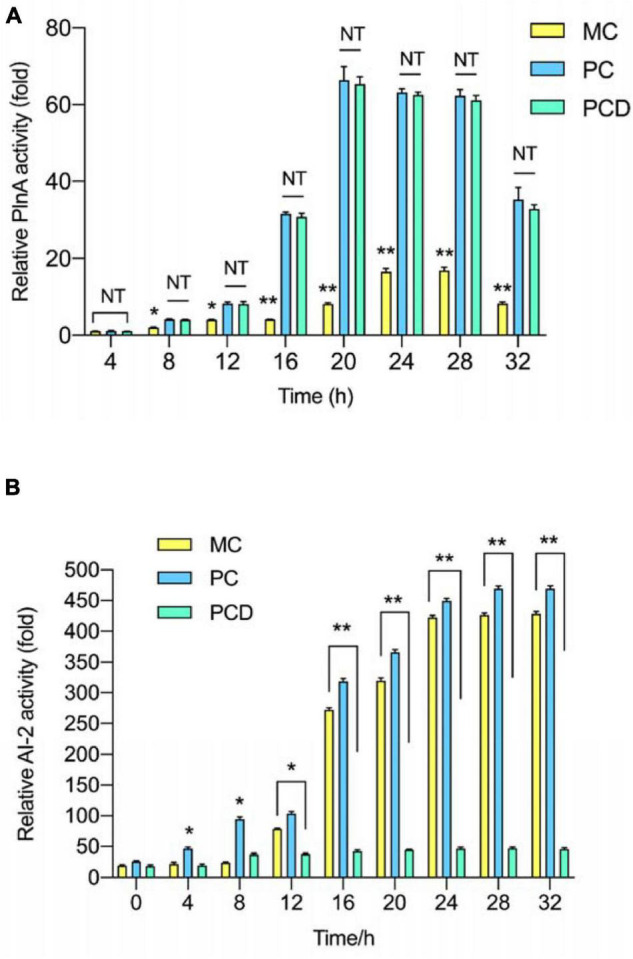
QS signal molecules activity in co-culture. **(A)** Relative PlnA activity. **(B)** Relative AI-2 activity. PC means positive bacteriocin-inducing co-culture of *L. plantarum* RX-8 and *B. subtilis* BS-15 (10^6^:10^6^ CFU/ml) at 37°C in 4–32 h. PCD means PC with 200 mM D-ribose. MC means monoculture of *L. plantarum* RX-8 (10^6^ CFU/ml) at 37°C in 4–32 h. NT indicates that there were no significant differences in the results of control; *p < 0.05 and **p < 0.01.

PlnA-inducing activity and AI-2 activity in monoculture (MC) and co-culture (PC and PCD) were analyzed every 4 h. As shown in Figure 6A, the PlnA-inducing activity in all treatments were increased from 8 to 20 h, peaked at 20 h, followed by a sudden decrease at 32 h. Combined with the results in Figure 5, the changing tendency of PlnA activity in MC, PC, and PCD was consistent with the variety of cell counts of *L. plantarum* RX-8 from 4 to 32 h. As a signal molecule of intraspecies QS system, the concentration of PlnA was enhanced with the increase in viable cell counts. Differently, the PlnA-inducing activity was dramatically increased in PC and PCD, whereas the viable cell counts of *L. plantarum* RX-8 were slightly increased compared with that in MC. Hence, it can be deduced that *B. subtilis* BS-15 probably produces some certain compounds, which could promote PlnA secretion of *L. plantarum* RX-8. PlnA-inducing activity was no difference in PC and PCD, while it was greater in PC and PCD than in MC from 8 to 32 h. These results suggested that more PlnA in co-culture was secreted to enhance plantaricin production, and inhibiting AI-2 activity would not influence PlnA-induced intraspecies communication. As shown in Figure 6B, AI-2 activity in MC and PC steadily increased from 4 to 32 h, while the AI-2 activity maintained a low level stably owing to the presence of D-ribose.

### Effects of *Bacillus subtilis* BS-15 on the Transcription of Plantaricin Biosynthesis and Autoinducer-2 Synthesis-Related Gene

**FIGURE 7:**
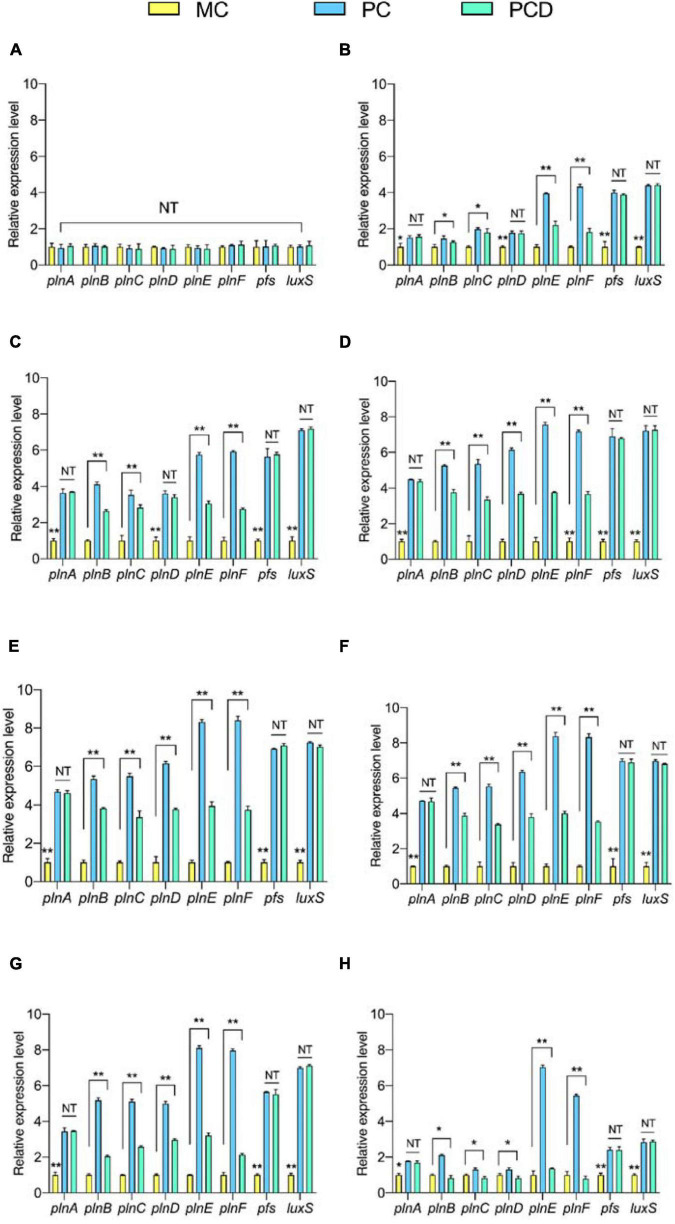
The relative transcriptional levels of genes *plnABCDEF*, *pfs*, and *luxS* at 4 h **(A)**, 8 h **(B)**, 12 h **(C)**, 16 h **(D)**, 20 h **(E)**, 24 h **(F)**, 28 h **(G)**, and 32 h **(H)**, respectively. The yellow bar (left) means MC of *L. plantarum* RX-8 (10^6^ CFU/ml) without *B. subtilis* BS-15 at 37°C in 4∼32 h, the blue bar (middle) means PC, of positive bacteriocin-inducing co-culture of *L. plantarum* RX-8 and *B. subtilis* BS-15 (10^6^:10^6^ CFU/ml) at 37°C in 4–32 h, and the green bar (right) means PCD, PC with 200 mM D-ribose. NT indicates that there were no significant differences in the results of the control; *p < 0.05 and **p < 0.01.

To explore why *B. subtilis* BS-15 could enhance the biosynthesis of plantaricin (Figure 7), the genes of *plnABCDEF*, *pfs*, and *luxS* in *L. plantarum* RX-8 were determined by PCR (Supplementary Figure 1). Meanwhile, these genes were detected at the transcriptional level in PC, PCD, and MC. According to Figure 7, the transcriptional levels of *plnE* and *plnF* in co-culture significantly increased in 8–32 h compared with monoculture (*p* < 0.05), and the highest values of 8.32-fold (*plnE*) and 8.40-fold (*plnF*) in PC were detected at 20 h. A similar tendency was observed in co-culture plantaricin production. Moreover, the expression of *plnABCD*, *pfs*, and *luxS* was positively correlated with plantaricin production in co-culture, indicating that *B. subtilis* BS-15 probably induced plantaricin effect by upregulating the expression of these genes.

In contrast to the decreasing trend found for interspecies QS signal AI-2 activity after the addition of D-ribose, the expression levels of *pfs* and *luxS* in PCD were considerably upregulated (*p* < 0.05). This fully verified D-ribose inhibits AI-2 by competitive binding to specific receptors rather than influencing the expression of key AI-2 biosynthesis enzymes (LuxS and Pfs). While 92.76% of AI-2 activity was lost following the addition of 200 mM D-ribose, the relative expression of *plnE* and *plnF* declined by only 48.02 and 42.53%, and the plantaricin production was decreased by only about 50% in PCD at 20 h. Additionally, similar changes in *plnBCD* transcriptional levels were observed in PCD. These findings suggested that the AI-2-mediated interspecies QS system had a role in the regulation of plantaricin induction when co-cultured with *B. subtilis* BS-15, but it was not exclusive to induction.

Given that the transcriptional levels of *plnA* and *plnEF* in co-culture were elevated even after the addition of D-ribose, it was hypothesized that *B. subtilis* BS-15 probably activated the *plnA* transcription by secreting a specific metabolite and that the PlnA-mediated intraspecies QS system played an important role in the plantaricin induction in co-culture. There was no significant difference in *plnA* transcriptional level between PC and PCD at all tested times (*p* > 0.05), indicating that the inducing activities of PlnA and AI-2 were independent.

### Proteomics Analysis

**FIGURE 8:**
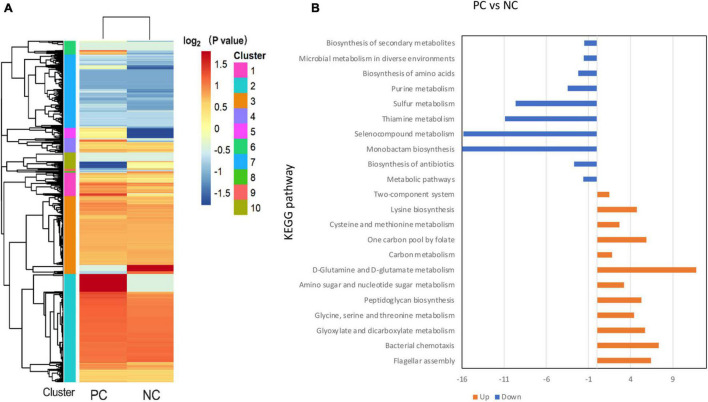
Categorical proteomics summary of *B. subtilis* BS-15 in co-culture. **(A)** Heatmap of different identified proteins. **(B)** Identified proteins were mapped to different KEGG pathways. PC means positive bacteriocin-inducing co-culture of *L. plantarum* RX-8 and *B. subtilis* BS-15 (10^6^:10^6^ CFU/ml) at 37°C for 20 h, and NC means negative bacteriocin-inducing co-culture of *L. plantarum* RX-8 and *B. subtilis* BS-15 (10^6^:10^4^ CFU/ml) at 37°C for 20 h.

The label-free proteomics analysis detected 211 proteins from *B. subtilis* BS-15, which were significantly (*p* < 0.05) different in PC and NC at 20 h. Meanwhile, the expression of 165 proteins was upregulated, whereas 46 proteins were downregulated (Figure 8A). The 211 differentially expressed proteins were functionally identified using the GO category system and KEGG pathway analysis, with the findings displayed in Figure 8B and Supplementary Figure 2.

To be more precise, these up-regulated proteins are mainly involved in flagellar assembly (MotB, FliM, FliG, FliY, and CheA), anaerobic respiration (CydA, CydB, and NarH), peptidoglycan (PG) biosynthesis (MurB, MurC, and MurD), and glycine cleavage system (GCS) (GcvPA, GcvPB, GcvH, GcvT, and PurU). In terms of the flagellar assembly pathway, CheA is a core protein of chemotaxis in *B. subtilis* that senses the chemical environment (Arapov et al., 2020), while MotB, FliM, FliG, and FliY control the direction of flagellar rotation based on chemotaxis levels (Phillips et al., 2015). It is likely that *B. subtilis* BS-15 dodged attack under PC by changing the directions of flagellar rotation, thus, moving to more favorable conditions. In the case of anaerobic respiration, CydA and CydB would be active only in insufficient oxygen (Larsson et al., 2005). NarH, a subunit of respiratory nitrate reductase, is required for the use of nitrate (NO_3_^–^) or nitrite (NO_2_^–^) as electron acceptors in low oxygen availability (Sonenshein et al., 2002). As a result, it is likely that *B. subtilis* BS-15 in PC survived by adapting its respiration pathway in response to environment changes. The upregulation of MurB, MurC, and MurD at protein level suggested that there were more PG in PC than in NC, as these proteins are involved in the synthesis of PG precursors (Real and Henriques, 2006; Vasudevan et al., 2007). The GCS catalyzed the oxidative decarboxylation of glycine and was dependent on tetrahydrofolate (THF) biosynthesis (Yang et al., 2020). It is helpful to obtain energy and 5,10-methylene-tetrahydrofolate, a C1 unit that is necessary for the production of serine, thymidine, and purines (Kikuchi et al., 2008). Almost every protein in the GCV system showed high upregulation, and PurU, a key enzyme of THF in PC, also increased. It can be deduced that the PC induces the GCS system. The functions of downregulated proteins mainly gathered in thiamin pyrophosphate biosynthesis (ThiC and ThiG), chorismate mutase (AroH), and ATP-sulfurylase (Sat). These pathways are associated with energy metabolism (Simonics et al., 2002; Pratap et al., 2017), indicating that *B. subtilis* BS-15 in NC may have been subjected to energy limitation.

## Discussion

Plantaricin is a broad-spectrum bacteriocin produced by *L. plantarum* RX-8 with significant food industry application potential to control foodborne pathogens. Low plantaricin production has been considered a major limitation restricting its commercialization. Considering induction of bacteriocin production by co-culture is widespread among plantaricin-producing *L. plantarum* strains, co-culturing with specific bacterial strains as an environmental stimulus may be a promising strategy to improve plantaricin production in food industry (Chanos and Mygind, 2016). In our study, co-cultured plantaricin production was 32 times higher than the plantaricin generated by the monoculture of *L. plantarum* RX-8. To facilitate the screening of effective bacteriocin-inducer strains and promote large-scale production and application of plantaricin in the food industry, preliminary study on the induction regulation mechanism of bacteriocin production by co-culture was carried out.

In the present study, *B. subtilis* BS-15 enhanced the plantaricin production of *L. plantarum* RX-8 without cell-to-cell physical contact between the two strains. A similar finding was reported in *L. plantarum* DC400, where bacteriocin increased due to the co-culturing with *Fructilactobacillus sanfranciscensis* DPPMA174 or *Furfurilactobacillus rossiae* A7 in a double-culture vessel apparatus separated by a 0.4-μm membrane filter (Di Cagno et al., 2009). It is indicated that cell-to-cell direct physical contact in co-culture is not necessary for plantaricin-inducing effect.

Considering the complexity of the interactions between *L. plantarum* RX-8 and *B. subtilis* BS-15, the inoculum ratio of the two bacteria in co-culture was a critical parameter. As observed, different inoculum ratios may have a distinct effect on the induction of plantaricin production in co-culture, implying that the inoculum ratio in co-culture was a key parameter in bacteriocin-inducing activity. According to Ariana and Hamedi (2017), the nisin synthesis may be induced at three different inoculum ratios (1:1, 1:2 and 2:1) of *Yarrowia lipolytica* ATCC 18942 and bacteriocin-producing *Lactococcus lactis* subsp. *lactis* UTMC 106. The largest amount of nisin was produced at a 1:1 inoculum ratio (Ariana and Hamedi, 2017). The co-culture bacteriocin-producing *Lactobacillus delbrueckii* subsp. *bulgaricus* BB18 and *Streptococcus thermophilus* 11A had similar behaviors. Bacteriocin production was dependent on the initial ratio between the two microorganisms, and bacteriocin activity in the initial ratio of 1:1 increased by 36% compared with that of 3:1 (Simova et al., 2008).

In order to ascertain the regulation intensity of AIP-mediated interspecies QS system/AI-2-mediated intraspecies QS system on plantaricin induction and their relationship in co-culture, the study analyzed the effect of co-culturing with *B. subtilis* BS-15 on bacteriocin-producer *L. plantarum* RX-8 cell growth, plantaricin production, QS signal molecule PlnA/AI-2 secretion, as well as plantaricin biosynthesis gene cluster and AI-2 synthesis-related gene expression. Results indicated that while the viable cell counts of *L. plantarum* RX-8 were slightly higher in co-culture (PC) than in monoculture (MC), the bacteriocin activity, PlnA inducing, and AI-2 activity were stronger in PC than in MC. It was concluded that there is a strong and positive correlation between QS signal molecule activity and plantaricin production in co-culture, but the correlation between viable cell counts and plantaricin production was not apparent. According to Man et al. (2014), when *L. plantarum* KLDS1.0391 was co-cultured with *Lactobacillus helveticus* KLDS1.9207, cell numbers were slightly higher than in monoculture, whereas the AI-2 activity was enhanced effectively (Man et al., 2014). A similar relationship between PLNC8 activity and cell number of *L. plantarum* NC8 was observed in co-culture with inducer strains (Maldonado et al., 2004). Furthermore, when AI-2 inhibitor was added, bacteriocin activity decreased in comparison with that in PC. As a result, it was found that AI-2 was involved in the bacteriocin-inducing activity of *B. subtilis* BS-15 for *L. plantarum* RX-8. Similar findings were observed in the co-culture of *L. plantarum* KLDS1.0391 and *L. helveticus* KLDS1.9207 (Man et al., 2014), as well as in *L. plantarum* DC400 that was co-cultured with *F. sanfranciscensis* DPPMA174 or *F. rossiae* A7 (Di Cagno et al., 2009). In accordance with the bacteriocin activity, the PlnA-inducing activity was dramatically increased in PC and PCD compared with MC. Like AI-2, PlnA is a crucial parameter for co-culture-induced bacteriocin synthesis. When co-culturing with another type of AIP, PlNC8 had the same effect (Maldonado et al., 2004). Moreover, PlnA-inducing activity in PC is not greatly different from that in PCD (*p* > 0.05). A similar pattern was also observed at the transcriptional level of the *plnA* gene. That indicated that AI-2 inhibitor had no effect on PlnA production in co-culture. Therefore, the inducing impact of PlnA and AI-2 was independent in co-culture. Interestingly, the PCD massively boosted the PlnA-inducing activities, while the viable cell counts of *L. plantarum* RX-8 only increased marginally. Apart from the effect of viable cell counts, it can be deduced that *B. subtilis* BS-15 likely produces some compounds that promote the secretion of PlnA of *L. plantarum* RX-8. In the case of Paracin 1.7, the CFS of a *B. subtilis* strain could improve the output of *Lacticaseibacillus paracasei* HD17 of Paracin 1.7 in co-culture, and the active compounds in the *B. subtilis* culture supernatant were several peptides between 15 and 45 kDa (Ge et al., 2014). Due to the fact that the inducing compounds from *B. subtilis* BS-15 have not been discovered or defined, further research will be required. The findings related to plantaricin biosynthesis gene cluster and AI-2 synthesis-related gene expression have further proved the above findings.

It is hypothesized that the mechanism by which *L. plantarum* RX-8 response to intraspecific/interspecific QS signal molecules (PlnA/AI-2) could be two paths: One is that AI-2 activates the transcription of genes *plnBCD*, followed by an increase in the transcription of genes *plnEF*, resulting in a significant increase in plantaricin production. Another possibility is that a specific metabolite from *B. subtilis* BS-15 activated the transcription of *plnA*, and then more *PlnA* was released to enhance the transcription of the three-component regulatory system (coded by *plnBCD*) and finally led to a higher plantaricin production. Man et al. (2012, 2014) discovered that AI-2 acted as an indirect trigger of upregulation of genes *plNC8HK–plnD*, and enhance bacteriocin production in *L. plantarum* KLDS1.0391 (Man et al., 2012, 2014). The capability of exogenous bacteria to induce bacteriocin production is apparently very strain specific (Rojo-Bezares et al., 2007). Possibly, the active substance released by *B. subtilis* BS-15 might lose its inducing effect when it is co-cultured with other bacteriocin-producing strains.

The effect of varying inoculum ratios of *L. plantarum* RX-8 and *B. subtilis* BS-15 on plantaricin production showed that the proper inoculation of *B. subtilis* BS-15 was vital for a successful inducing effect in co-culture. This study carried out differential proteomics on *B. subtilis* BS-15 between PC and NC to explore how bacteriocin-inducing *B. subtilis* BS-15 activated PlnA/AI-2. Given the change in *B. subtilis* BS-15 at the protein level, it is likely that *B. subtilis* BS-15 in PC detected changes in the chemical environment before moving to more favorable conditions by changing the direction of flagellar motor rotation to avoid plantaricin attack (Cannistraro et al., 2011), thus, producing more PG to guarantee its survival (Larsson et al., 2005). Moreover, the terminal oxidases and other respiratory enzymes of *B. subtilis* BS-15 in PC were altered to conserve energy, and the GCV system generated ATP and NADH through utilizing glycine as a nitrogen source. Additionally, the GCV system is capable of generating C1 units, which are used to produce purines, histidine, thymine, and methionine (Kikuchi et al., 2008). It was hypothesized that activating the GCV system would disrupt a crucial function of *B. subtilis* BS-15, resulting in modification or synthesis of signal molecules that may regulate bacteriocin production of *L. plantarum* RX-8. It is required to be determined in future research through gene disruption mutants or metabolomics analysis. Due to the fact that the strongly downregulated proteins (thiamin pyrophosphate biosynthesis, chorismate mutase, and ATP-sulfurylase) were related to energy and metabolic pathways, it could be concluded that *B. subtilis* BS-15 in NC lacked conserving energy and suffered from thiamin pyrophosphate and energy shortage.

## Conclusion

The co-culturing of *B. subtilis* BS-15 has been proven to be an alternative for the induction of plantaricin production. When *L. plantarum* RX-8 and *B. subtilis* BS-15 were co-inoculated in MRS for 20 h at 1:1 inoculum ratio (10^6^:10^6^ CFS/ml), plantaricin achieved the largest output (2048 AU/ml) in co-culture. In comparison with the monoculture of *L. plantarum* RX-8, plantaricin production in co-culture with *B. subtilis* BS-15 improved by 32 times. Both the PlnA-mediated intraspecies QS system and the AI-2-mediated interspecies QS system play important roles in plantaricin induction when co-culturing with *B. subtilis* BS-15, and the inducing effect of PlnA and AI-2 was independent in co-culture. It is possible that AI-2 activates the transcription of plantaricin biosynthesis genes, resulting in a significant increase in plantaricin production. Meanwhile, the inducing strain BS-15 appears to activate the secretion of PlnA, triggering the QS system, which further increases plantaricin production. Differential proteomics analysis of *B. subtilis* BS-15 in co-culture indicated that the bacteriocin-inducing regulatory mechanism might be related to flagellar assembly, peptidoglycan biosynthesis, anaerobic respiration, glycine cleavage system, and thiamin pyrophosphate biosynthesis. We are currently performing a detailed study on the bacteriocin-inducing regulatory mechanism in co-culture, using integrated transcriptomic and proteomic methods.

## Data Availability Statement

The original contributions presented in the study are included in the article/Supplementary Material, further inquiries can be directed to the corresponding author.

## Author Contributions

GL conceptualized the idea, made corrections, and approved the research article for publication. XL, YS, and JZ performed the experiments. RN, XH, and JD analyzed the results and wrote the first draft of the manuscript. YL and RN revised the article. All authors contributed to the article and approved the submitted version.

## Conflict of Interest

The authors declare that the research was conducted in the absence of any commercial or financial relationships that could be construed as a potential conflict of interest.

## Publisher’s Note

All claims expressed in this article are solely those of the authors and do not necessarily represent those of their affiliated organizations, or those of the publisher, the editors and the reviewers. Any product that may be evaluated in this article, or claim that may be made by its manufacturer, is not guaranteed or endorsed by the publisher.

## Footnotes

1
https://portal.genego.com

2
https://www.genome.jp/kegg/
