# Supplementary figures and images for: Bacillus subtilis BS-15 Effectively Improves Plantaricin Production and the Regulatory Biosynthesis in Lactiplantibacillus plantarum RX-8
Source: Front Microbiol. 2022 Jan 28;12:772546. doi: 10.3389/fmicb.2021.772546 (PMC8837263; doi:10.3389/fmicb.2021.772546)

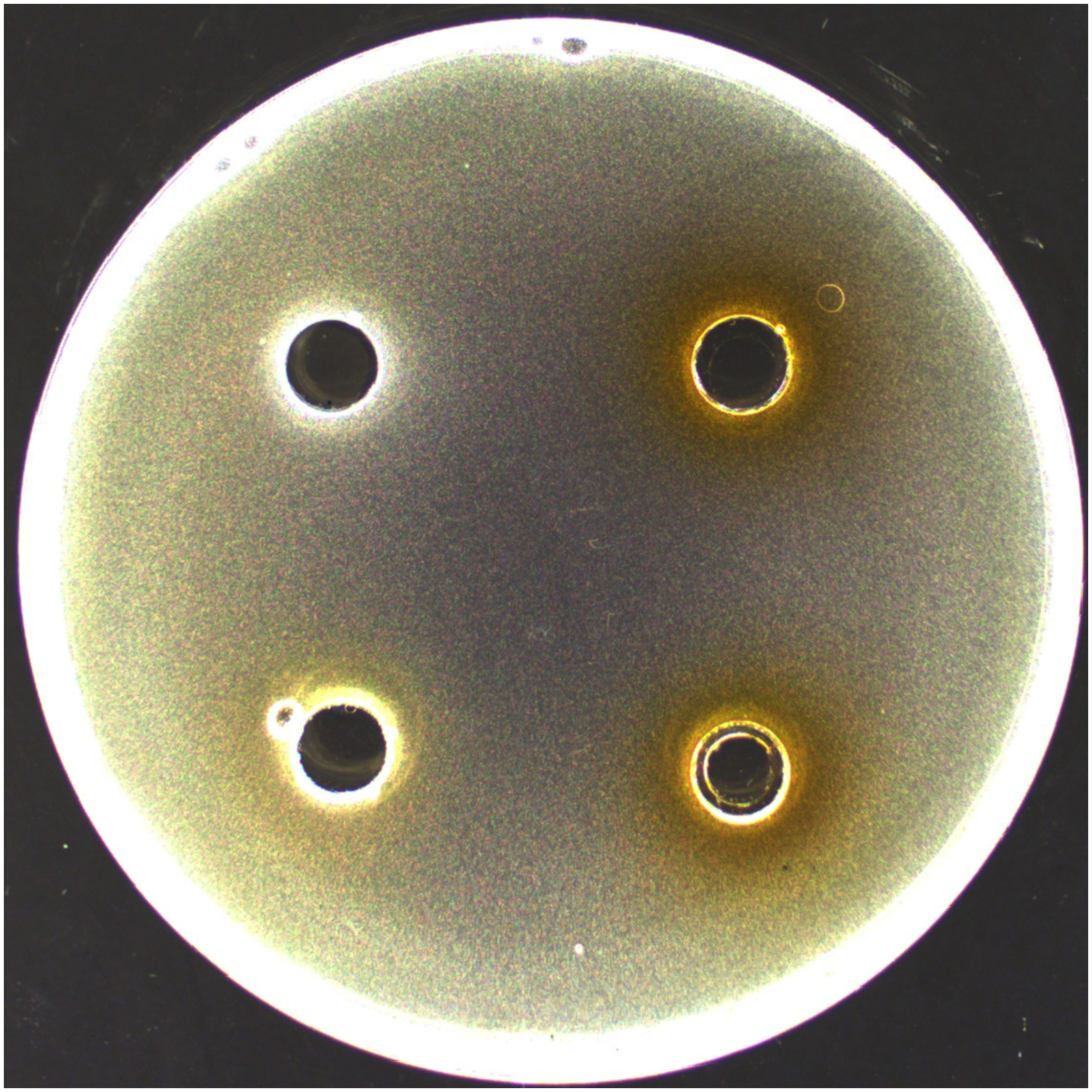

Supplement: Supplementary file 1 [file Data_Sheet_1.ZIP › 4h.tiff]

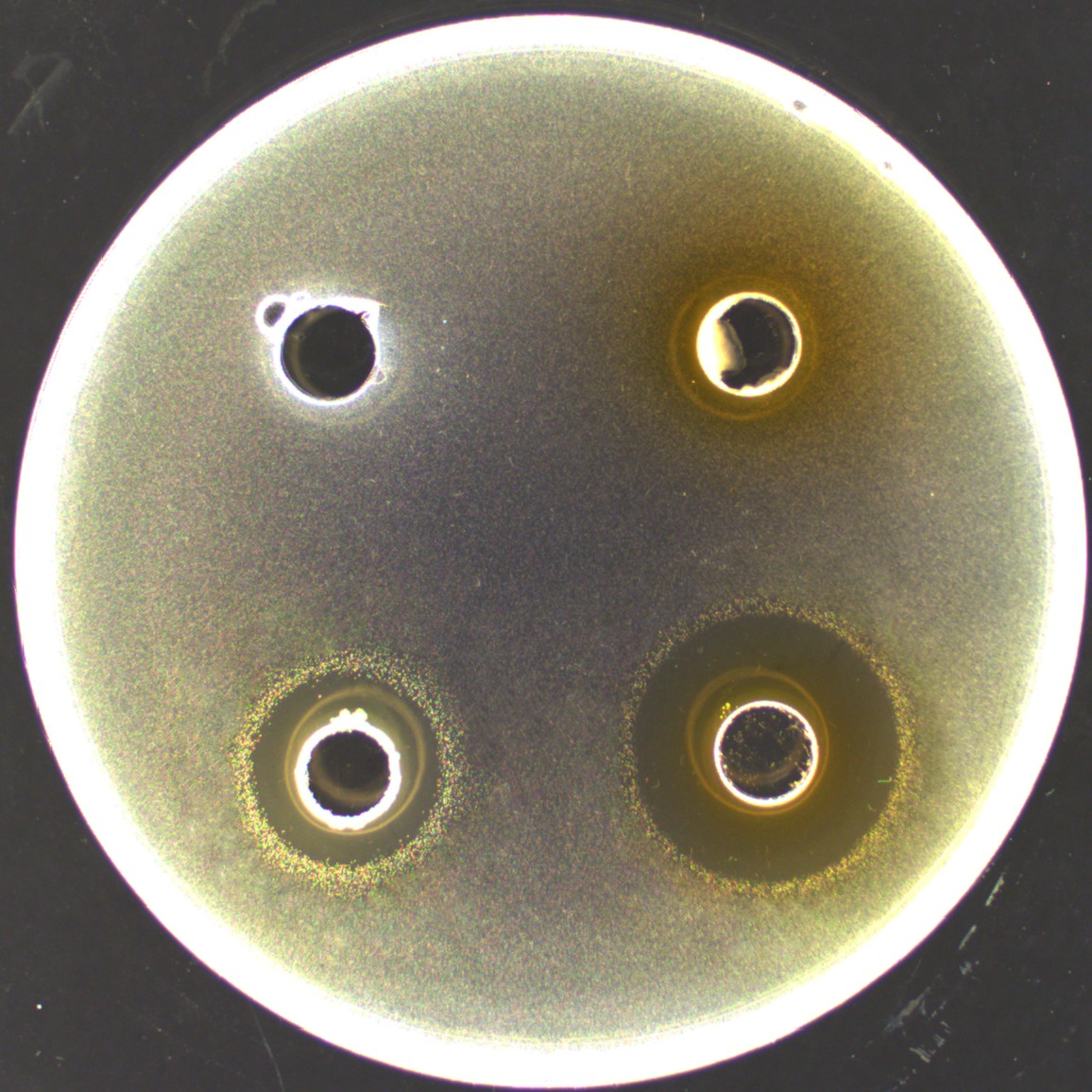

Supplement: Supplementary file 1 [file Data_Sheet_1.ZIP › 8h.tiff]

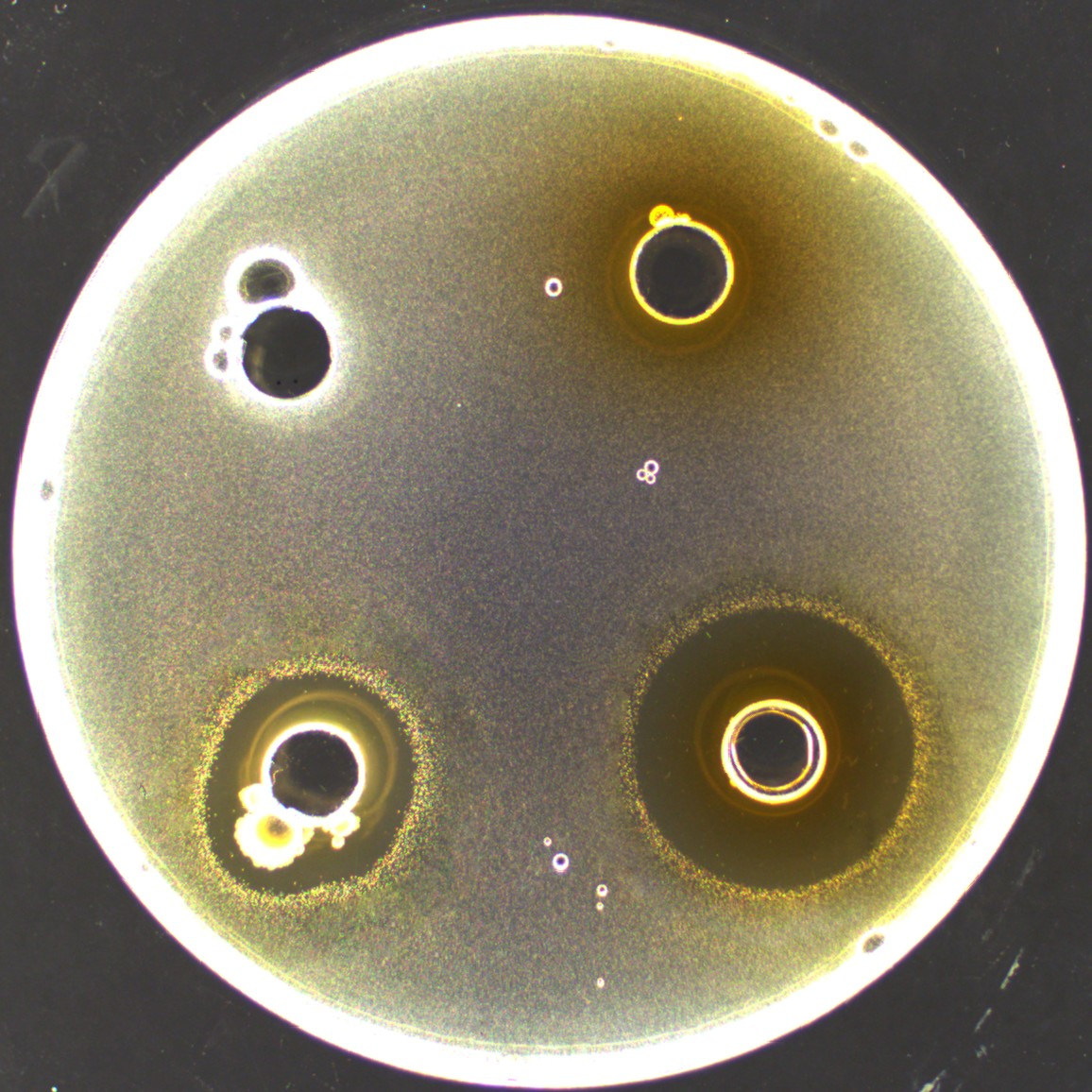

Supplement: Supplementary file 2 [file Data_Sheet_2.ZIP › 16h.tiff]

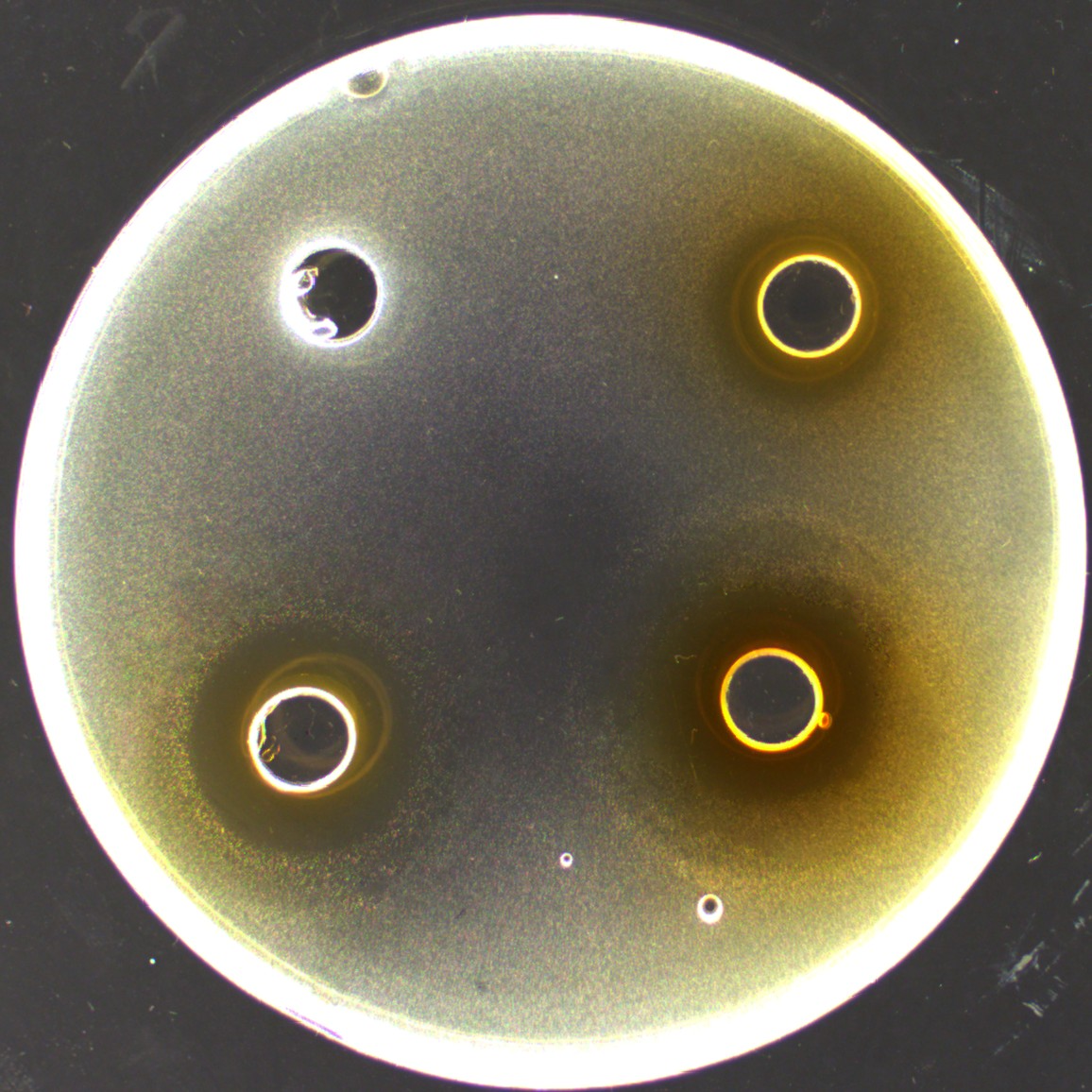

Supplement: Supplementary file 2 [file Data_Sheet_2.ZIP › 20h.tiff]

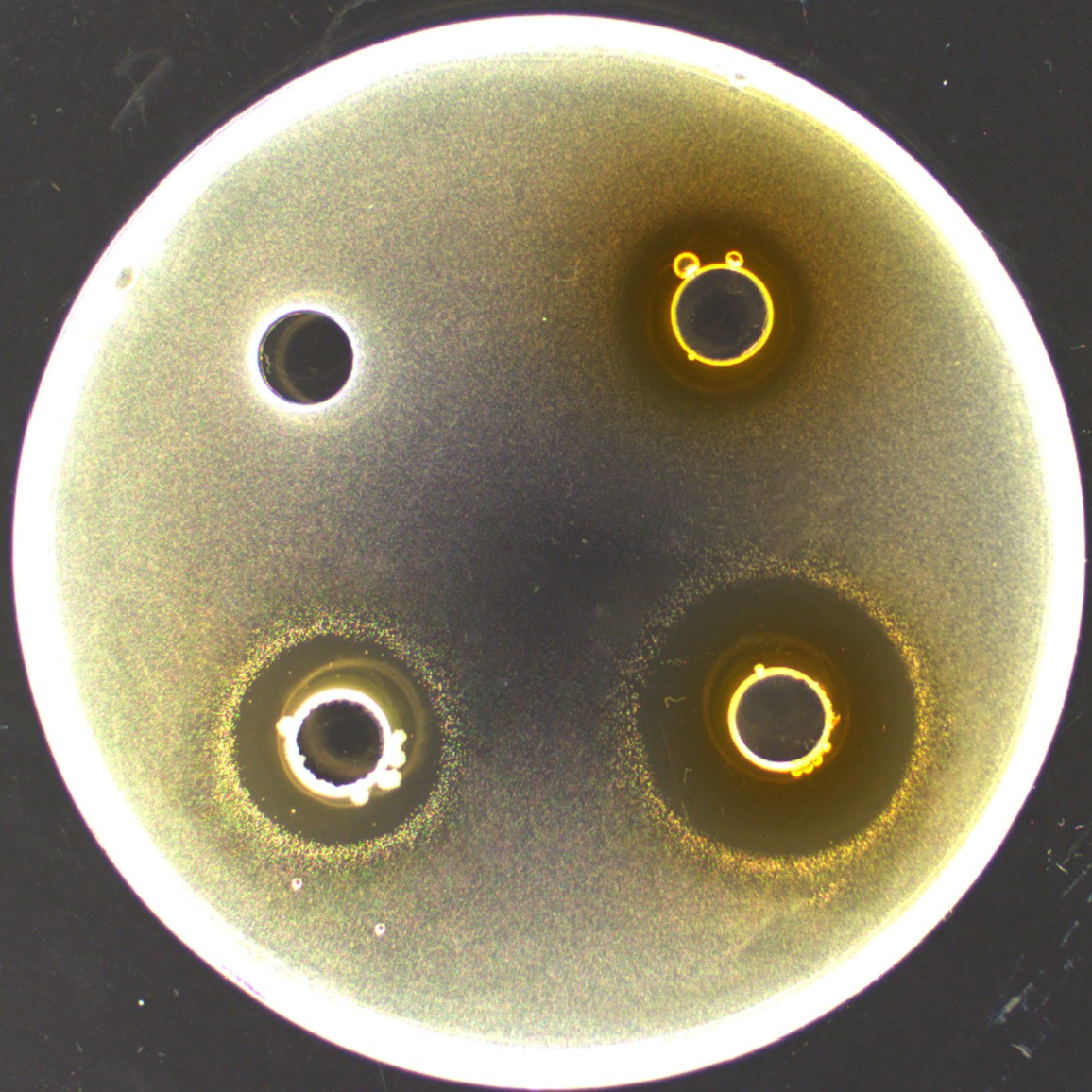

Supplement: Supplementary file 2 [file Data_Sheet_2.ZIP › 24h.tiff]

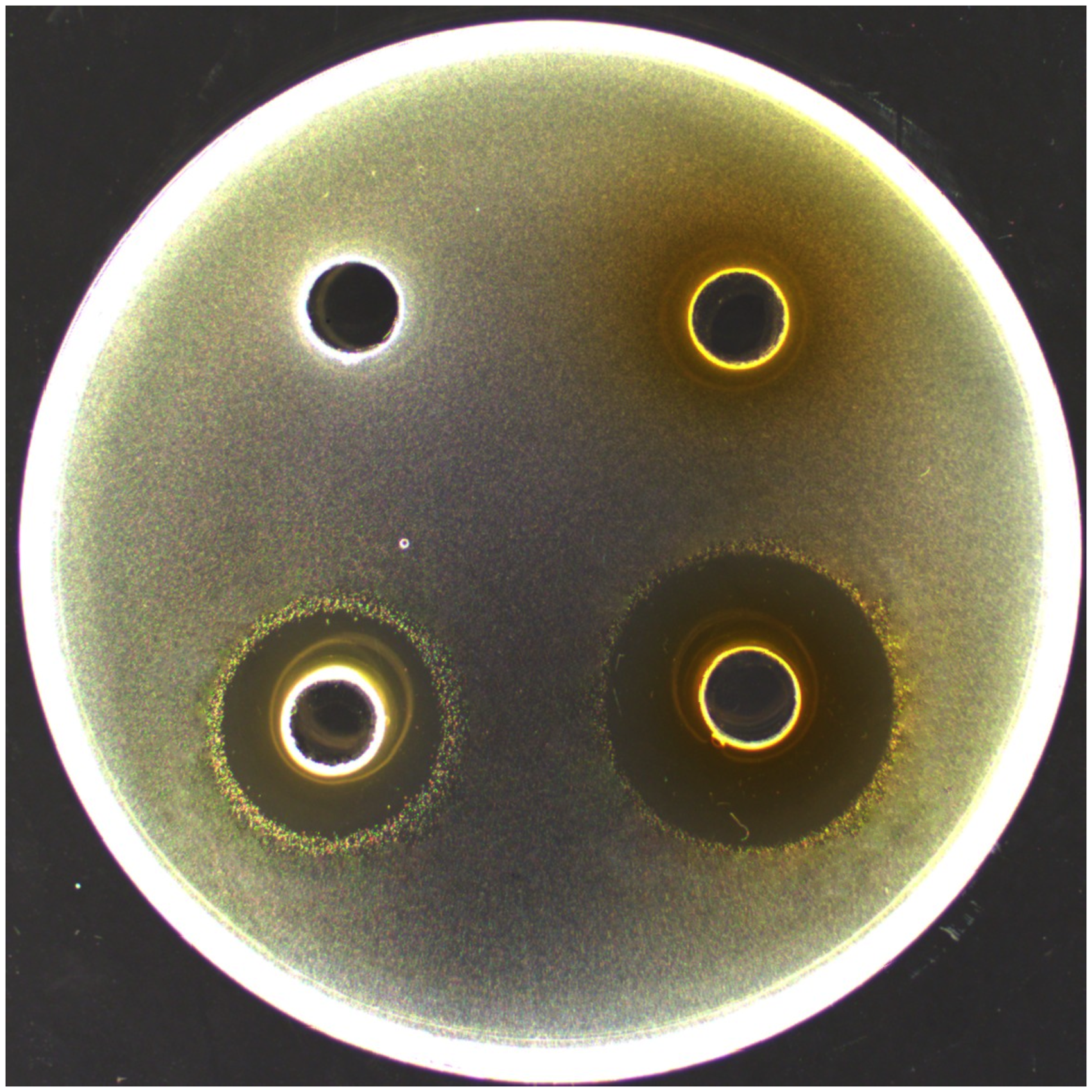

Supplement: Supplementary file 2 [file Data_Sheet_2.ZIP › 12h.tiff]

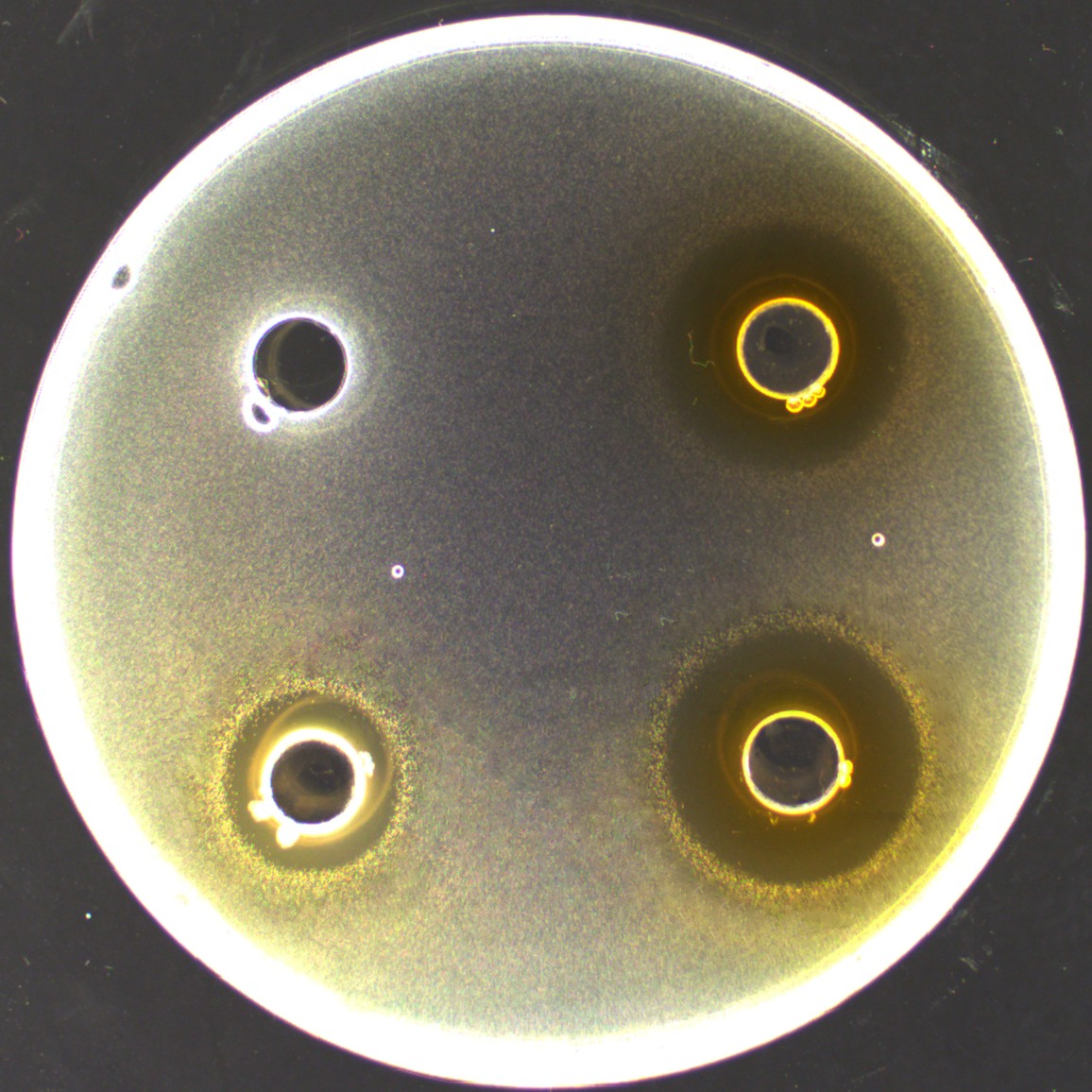

Supplement: Supplementary file 3 [file Data_Sheet_3.ZIP › 32h.tiff]

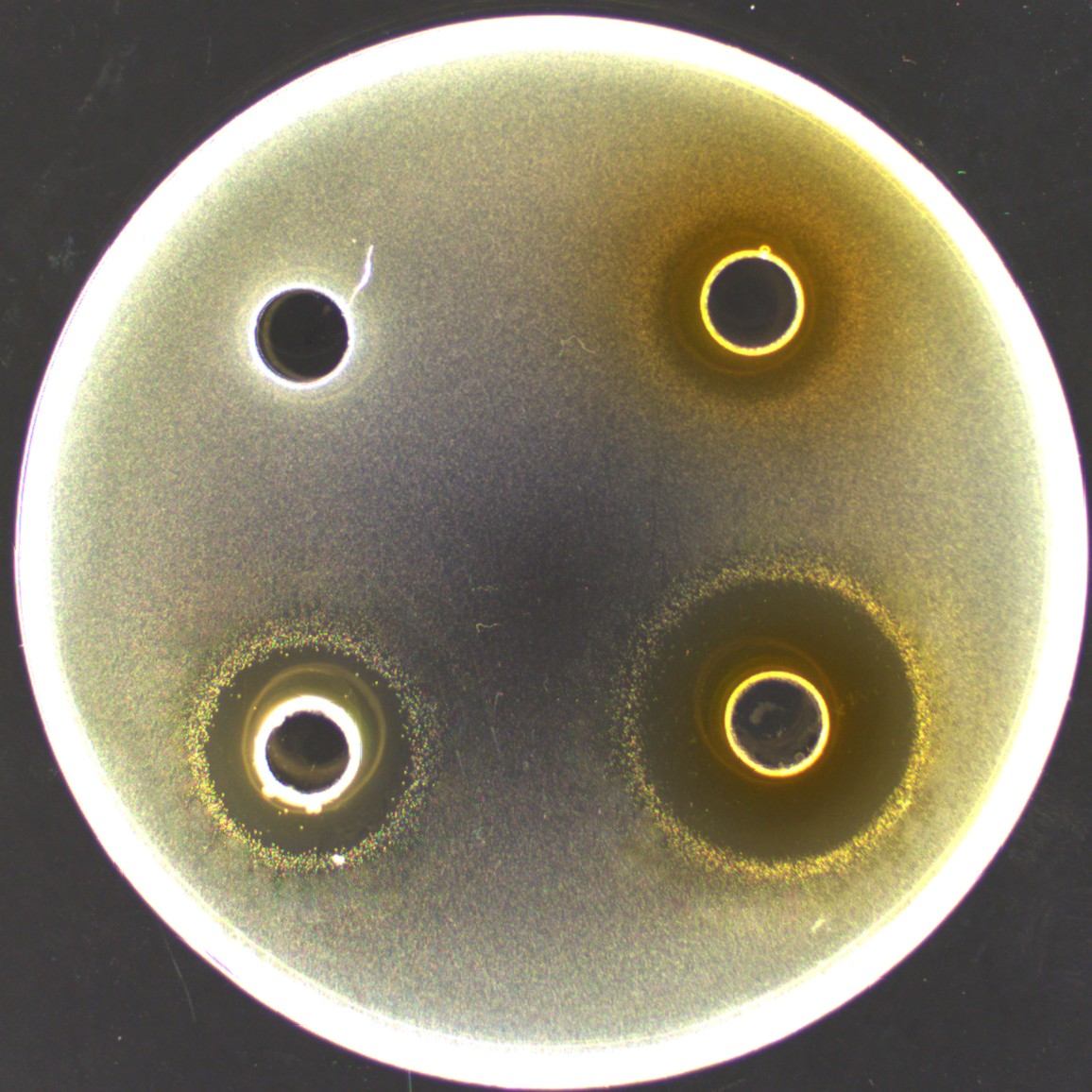

Supplement: Supplementary file 3 [file Data_Sheet_3.ZIP › 28h.tiff]

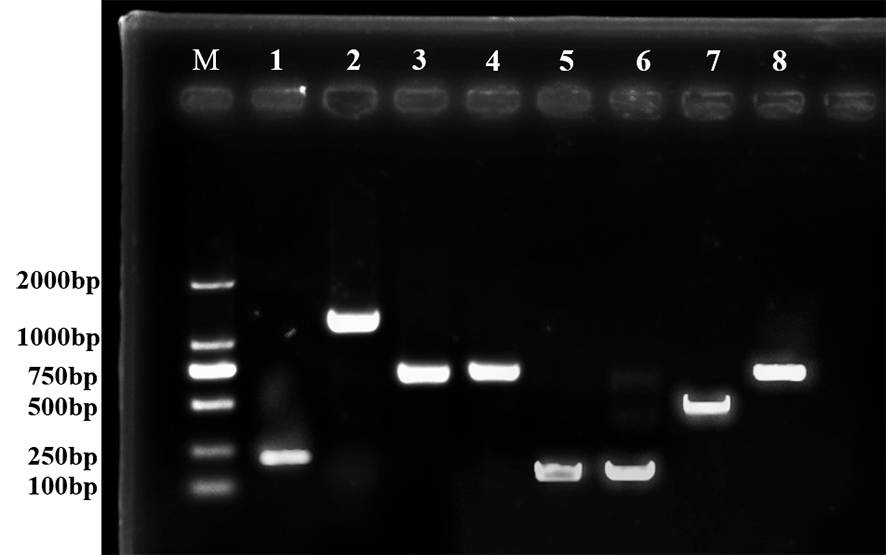

Supplement: Supplementary file 4 [file Image_1.TIF]

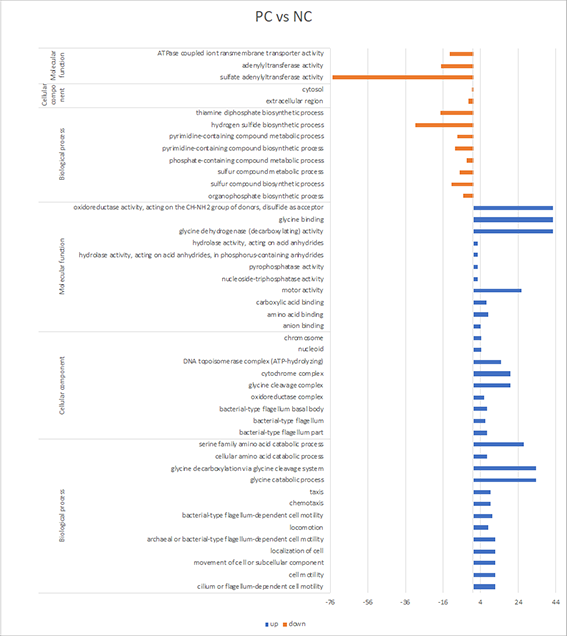

Supplement: Supplementary file 5 [file Image_2.TIF]

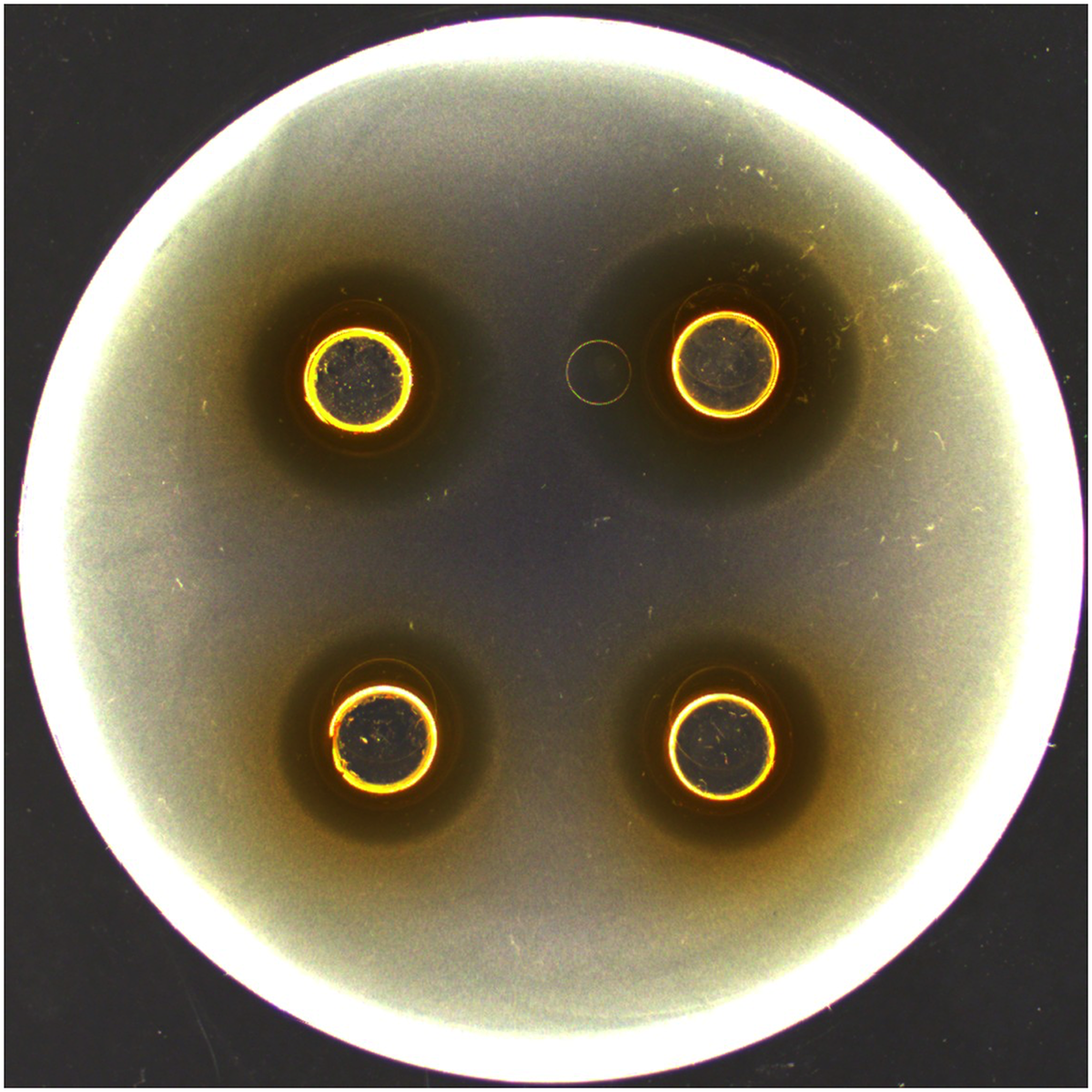

Supplement: Supplementary file 6 [file Image_3.TIFF]
