# Supplementary material for: Bacillus subtilis BS-15 Effectively Improves Plantaricin Production and the Regulatory Biosynthesis in Lactiplantibacillus plantarum RX-8
Source: Front Microbiol. 2022 Jan 28;12:772546. doi: 10.3389/fmicb.2021.772546 (PMC8837263; doi:10.3389/fmicb.2021.772546)
Supplement: Supplementary file 18 [file Table_12.DOCX]

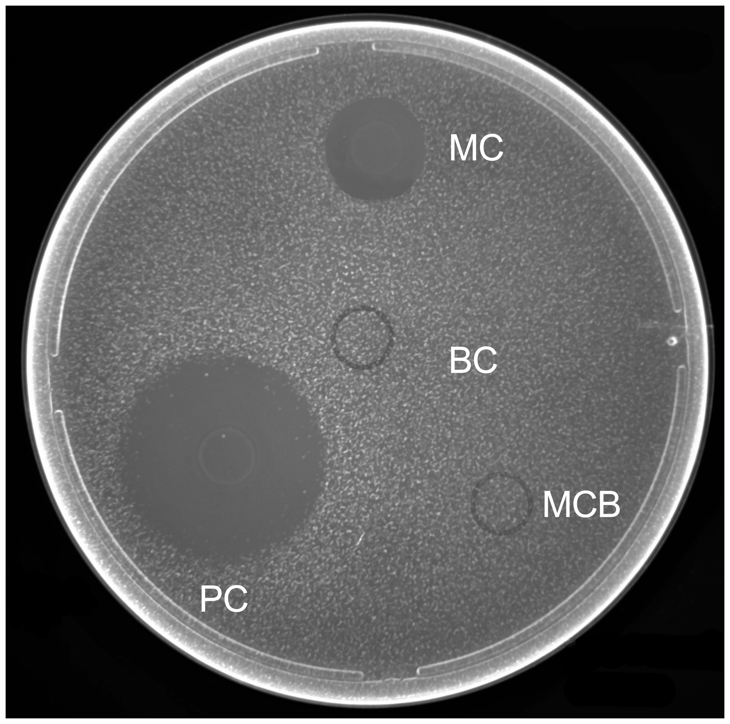


**Supplementary Figure 3 Antimicrobial activity of CFSs in co-culture and mono-culture.** PC means positively bacteriocin-inducing co-culture of *L. plantarum* RX-8 and *B. subtilis* BS-15 (10^6^:10^6^ CFU/mL) in MRS at 37°C for 24 h. MC means mono-culture of *L. plantarum* RX-8 (10^6^ CFU/mL) in MRS at 37°C for 24 h, MCB means mono-culture of *B. subtilis* BS-15 (10^6^ CFU/mL) in MRS at 37°C for 24 h. BC means blank control (20 mM sodium phosphate buffer, pH 6.0).
